# Increased Interleukin 18-Dependent Immune Responses Are Associated With Myopericarditis After COVID-19 mRNA Vaccination

**DOI:** 10.3389/fimmu.2022.851620

**Published:** 2022-02-18

**Authors:** Taejoon Won, Nisha Aggarwal Gilotra, Megan Kay Wood, David Matthew Hughes, Monica Vladut Talor, Jana Lovell, Aaron Michael Milstone, Charles Steenbergen, Daniela Čiháková

**Affiliations:** ^1^ Department of Pathology, Johns Hopkins University School of Medicine, Baltimore, MD, United States; ^2^ Division of Cardiology, Department of Medicine, Johns Hopkins University School of Medicine, Baltimore, MD, United States; ^3^ W. Harry Feinstone Department of Molecular Microbiology and Immunology, Johns Hopkins University Bloomberg School of Public Health, Baltimore, MD, United States; ^4^ Department of Chemical and Biomolecular Engineering, Johns Hopkins University Whiting School of Engineering, Baltimore, MD, United States; ^5^ Division of Pediatric Infectious Diseases, Department of Pediatrics, Johns Hopkins University School of Medicine, Baltimore, MD, United States

**Keywords:** interleukin 18, NLRP3 inflammasome, NK cells, Th1-type immune response, myopericarditis, COVID-19 mRNA vaccine

## Abstract

Myocarditis and myopericarditis may occur after COVID-19 vaccination with an incidence of two to twenty cases per 100,000 individuals, but underlying mechanisms related to disease onset and progression remain unclear. Here, we report a case of myopericarditis following the first dose of the mRNA-1273 COVID-19 vaccine in a young man who had a history of mild COVID-19 three months before vaccination. The patient presented with chest pain, elevated troponin I level, and electrocardiogram abnormality. His endomyocardial biopsy revealed diffuse CD68^+^ cell infiltration. We characterized the immune profile of the patient using multiplex cytokine assay and flow cytometry analysis. Sex-matched vaccinated individuals and healthy individuals were used as controls. IL-18 and IL-27, Th1-type cytokines, were highly increased in the patient with COVID-19 vaccine-related myopericarditis compared with vaccinated controls who experienced no cardiac complications. In the patient, circulating NK cells and T cells showed an activated phenotype and mRNA profile, and monocytes expressed increased levels of IL-18 and its upstream NLRP3 inflammasome. We found that recombinant IL-18 administration into mice caused mild cardiac dysfunction and activation of NK cells and T cells in the hearts, similar to the findings in the patient with myopericarditis after COVID-19 mRNA vaccination. Collectively, myopericarditis following COVID-19 mRNA vaccination may be associated with increased IL-18-mediated immune responses and cardiotoxicity.

## Introduction

Recently, myocarditis and myopericarditis cases following COVID-19 vaccination were reported in the United States and other countries (1–3). The incidence was two to twenty cases per 100,000 persons who have received at least one dose of any COVID-19 vaccines, with the potential risk increased in individuals who received 2^nd^ dose of mRNA-based vaccine (3, 4). Males between the ages of 16 and 29 years showed a higher incidence than other sex or age groups (3). In some case reports, a considerable proportion of patients had a history of COVID-19 before they received the vaccine (2, 5, 6). Most of the patients reported presented with chest pain, elevated cardiac troponin level, abnormal electrocardiogram (ECG) with diffuse ST-segment elevations, and slightly decreased left ventricular ejection fraction (2, 5–8). The diagnosis of myocarditis or myopericarditis was usually confirmed by cardiac magnetic resonance imaging (CMR) rather than an endomyocardial biopsy (9). Similar to myocarditis cases, acute pericarditis is suspected once patients have chest pain and abnormalities on ECG, echocardiogram, or CMR after COVID-19 vaccination (9). Pericarditis following COVID-19 vaccine developed in older patients later after vaccination compared with myocarditis or myopericarditis cases (4). The majority of implicated cases of COVID-19 vaccine-related myocarditis have been mild and self-limiting. Few rare deaths have been reported in more fulminant presentations (10–12).

Viral infection is the most common cause of myocarditis. Other infections, autoimmunity, toxins, medications, and drugs can also induce myocarditis (13, 14). Vaccine-related myopericarditis has been rarely reported in the past, primarily after live-attenuated smallpox vaccine in the military population (15). Rarely, the vaccination for influenza or hepatitis B has also been associated with myocarditis or myopericarditis (16). The pathogenic process of community-acquired myocarditis, commonly caused by a viral infection, is driven by activated immune cells, cytokines, and chemokines (17). Massive infiltration of T cells, myeloid cells, and eosinophils are histologically observed in the lymphocytic, giant cell, and eosinophilic myocarditis, respectively (18). In COVID-19 mRNA vaccine-related myocarditis, endomyocardial biopsy findings have varied. Some studies showed myocardial infiltration of lymphocytes, myeloid cells, or mixed immune cell populations, whereas others did not find significant myocardial inflammation (2, 5, 10–12). Meanwhile, autoantibodies against cardiac antigens have been detected in patients with community-acquired myocarditis, suggesting that activated B cells may play an important role in the initiation or progress of myocarditis (19). However, in a case study of myocarditis after the COVID-19 vaccine, no circulating autoantibodies related to myocarditis were detected (20).

IL-18 is an IL-1 superfamily cytokine that is primarily produced by monocytes and macrophages (21). For IL-18 production, its upstream signaling molecules such as NLRP3 inflammasome and caspase-1 are activated to cleave pro-IL-18 into its active form (22). As a Th1-type immune response inducer, IL-18 can activate NK cells, Th1 cells, and cytotoxic T lymphocytes to produce cytokines and effector molecules including IFN-γ, perforin, granzymes, or FasL (21). IL-18 is also known to play a pathogenic role in cardiovascular diseases. Circulating IL-18 levels are a prognostic marker associated with congestive heart failure, myocardial infarction, and cardiovascular death in patients with coronary heart disease (23–25). Supportively, IL-18 *in vivo* administration caused cardiac damage in mice by inducing myocardial hypertrophy, diastolic dysfunction, and extracellular matrix remodeling (26, 27).

Despite well-described clinical presentations, immune mechanisms driving myocarditis or myopericarditis after the COVID-19 mRNA vaccine are not fully understood. Here, we report a clinical case and comprehensive immune profile of a young male patient who had acute myopericarditis following the first dose of the mRNA-1273 COVID-19 vaccine (Moderna). We included controls who were vaccinated but did not have myocarditis to highlight how the immune responses in the patient with COVID-19 vaccine-related myopericarditis differ. We propose IL-18-mediated immune responses and cardiotoxicity as a possible mechanism of myopericarditis following COVID-19 mRNA vaccination.

## Materials and Methods

### Human Samples

We obtained plasma, PBMC, and endomyocardial biopsy from a patient with myopericarditis following COVID-19 mRNA vaccination (**Table 1**). The plasma and PBMC were collected on days 3 and 4 after vaccination, and the biopsy specimen was collected on day 3. As a control, the plasma and PBMC were obtained from five healthy controls and five individuals who received COVID-19 vaccine in the past 2 to 3 days (**Table 1**).

**Table 1:** Demographic and clinical information of the patient with myopericarditis after COVID-19 vaccine and controls.

Patient with myopericarditis after COVID-19 vaccine			
Sex	Male
Age (year)	24
COVID-19 vaccination	1^st^ dose of mRNA-1273
Days after vaccination	2
Body temperature (°C)	36.1
Blood pressure (mmHg)	125/94
Heart rate (beats per minute)	126
Oxygen saturation (%)	98
**Laboratory values**	**On admission**	**3-month follow-up**	**Reference range**
White blood cell count (K/mm^3^)	14.3	6.57	4.5-11.0
Absolute lymphocyte count (K/mm^3^)	0.8	1.19	1.1-4.8
Hemoglobin (g/dL)	13.8	15.7	13.9-16.3
Platelets (K/mm^3^)	178	212	150-350
Sodium (mmol/L)	139	–	135-148
Potassium (mmol/L)	4.3	–	3.5-5.1
Creatinine (mg/dL)	0.8	–	0.6-1.3
Urea nitrogen (mg/dL)	13	–	7-22
Aspartate aminotransferase (U/L)	116	–	0-37
Alanine aminotransferase (U/L)	35	–	0-40
Alkaline phosphatase (U/L)	35	–	30-120
Total bilirubin (mg/dL)	0.3	–	0.0-1.2
Troponin I (ng/mL)	0.47 (peak 46.0)	<0.04	<0.04
Pro-B-type natriuretic peptide (pg/mL)	390	–	0-125
Erythrocyte sedimentation rate (mm/h)	21	–	1-15
C-reactive protein (mg/dL)	8.4	0.9	<0.5
Lactate (mmol/L)	2.3	–	0.5-2.0
**Healthy controls (n=5)**			
Sex	Male
Age (year)	31.2 ± 3.6
COVID-19 vaccination	2^nd^ dose of BNT162b2
Days after vaccination	>180
**Vaccine controls (n=5)**			
Sex	Male
Age (year)	41.1 ± 9.4
COVID-19 vaccination	1^st^ dose of BNT162b2
Days after vaccination	2 ± 1.7

### Mice

Male A/J mice (000646) were purchased from the Jackson Laboratory. Mice were housed in specific pathogen-free animal facilities at the Johns Hopkins University School of Medicine. Experiments were conducted with 8- to 12-week-old age-matched mice. Mice were intraperitoneally injected with 1 μg of recombinant mouse IL-18 (BioLegend) in 100 μL PBS every other day for 8 days. On day 10, cardiac function was monitored using M-mode transthoracic echocardiography, and then mice were sacrificed. To isolate single cells, hearts were perfused with PBS for 3 minutes, minced using razor blades, and incubated in 5 mL tissue digestion enzyme solution for 30 minutes at 37°C with agitation. The digestion enzyme solution was prepared with 600 U/ml Collagenase (Worthington), 100 U/ml DNase I (Worthington), and 100 U/ml Hyaluronidase (Sigma). Cells were washed and filtered through 40 μm cell strainers (Falcon).

### Immunohistochemistry Staining

The biopsy tissue slides were deparaffinized, rehydrated, and blocked with Duel Endogenous Enzyme Block (Dako). Tissue sections were probed with primary antibody against CD68 (KP1, Abcam) or CD3 (SP7, Abcam) and then treated with HRP-conjugated secondary goat antibody against rabbit or mouse IgG (Leica). Mouse heart sections were stained with anti-NCR1 antibody (EPR23097-35, Abcam). The slides were counterstained with 50% hematoxylin in water.

### Multiplex Cytokine Assay

We used Milliplex Map Kit (EMD Millipore) to measure soluble factors in the plasma samples according to the manufacturer’s instructions. Briefly, after prewashing the plate, we added assay buffer, samples, and beads into wells and then incubated overnight at 4°C. After washing, detection antibodies were added, followed by streptavidin-phycoerythrin treatment. Plates were washed, filled with sheath fluid, and read on Luminex 200 (Luminex).

### Flow Cytometry

Human PBMC was stained with Live/Dead Fixable Aqua (Thermo Fisher) followed by blocking with TruStain FcX (BioLegend) and normal mouse serum (Thermo Fisher). Cells were surface-stained with fluorochrome-conjugated antibodies against CD45 (HI30), CD3 (UCHT1), CD19 (HIB19), CD4 (SK4), CD8 (RPA-T8), CD45RA (HI100), CCR7 (2-L1-A), CD38 (HB-7), CD27 (O323), IgD (IA6-2), CD56 (5.1H11), HLA-DR (G46-6), CD16 (3G8), CD14 (MΦP9), CD95 (eBioH4A3), CD107a (DX2), and PD-1 (EH12.2H7) (BD Biosciences, BioLegend, or Thermo Fisher). Cells were fixed, permeabilized with Cyto-Fast Fix/Perm Buffer (BioLegend), and stained with antibodies against intracellular antigens Ki-67 (Ki-67), IL-18 (74801), and NLRP3 (768319) (BioLegend or R&D Systems). For intracellular IL-18 and NLRP3 detection in monocytes, human PBMC was stimulated with lipopolysaccharides from *E. coli* O111:B4 (Sigma-Aldrich) in the presence of GolgiStop (BD Biosciences) for 4 hours at 37°C before staining. Single cells from mouse hearts were stained with Live/Dead Fixable Aqua, Fc-blocked with anti-CD16/CD32 monoclonal antibody (Thermo Fisher), and surface-stained with antibodies against CD45 (30-F11), NKp46 (29A1.4), CD11b (M1/70), CD3 (17A2), CD19 (1D3), CD4 (GK1.5), CD8 (53-6.7), Ly6G (1A8), F4/80 (BM8), CD64 (X54-5/7.1), Ly6C (HK1.4), CCR2 (SA203G11), CD44 (IM7), CD62L (MEL-14), and MHCII (M5/114.15.2) (BD Biosciences, BioLegend, or Thermo Fisher). Sample acquisition was performed on a BD FACSymphony flow cytometer (BD) running FACSDiva (BD). Results were analyzed using FlowJo software (BD).

### Real-Time RT-PCR

CD14^+^ monocytes or NK cells were enriched from PBMC by using CD14 Microbeads or NK Cell Isolation Kit (Miltenyi). Total RNA was extracted using RNeasy Plus Mini Kit (Qiagen). Single‐stranded cDNA was synthesized with iScript Reverse Transcription Supermix (Bio-Rad). Target genes were amplified using PowerUp SYBR Green PCR Master Mix (Thermo Fisher), and real-time cycle thresholds were detected *via* MyiQ2 thermal cycler (Bio-Rad). Data were analyzed by the 2^−ΔΔCt^ method and were normalized to *GAPDH* expression and then to biological controls. For SARS-CoV-2 RNA detection, total RNA was extracted from endomyocardial biopsy specimen using RNeasy Plus Mini Kit (Qiagen). Single-stranded cDNA was synthesized, and then SARS-CoV-2 S or N gene was amplified. Primers for SARS-CoV-2 N1, SARS-CoV-2 N2, and internal control RNase P were obtained from the Integrated DNA Technologies. According to the Centers for Disease Control and Prevention (CDC) guideline, a test is considered positive for SARS-CoV-2 if both SARS-CoV-2 N1 and N2 markers show Ct value < 40.

### Indirect Immunofluorescence Antibody Assay

We used Cardiac Muscle Antibody Test Kit (Scimedx) to detect autoantibodies against heart antigens in patient plasma. Monkey heart slides were covered with patient samples (1:10) or positive control antibody and then incubated for 30 minutes at room temperature in a humid chamber. After washing, slides were covered with FITC-conjugated human IgG antibody for 30 minutes and then read with a fluorescent microscope.

### ELISA

For anti-myosin S2-16 and S2-28 IgG ELISA, plates were coated with 0.5 µg/ml human cardiac myosin peptide S2-16 (KRKLEGDLKLTQESIMDLENDKQQL) or S2-28 (EKSEFKLELDDVTSNMEQIIKAKAN) overnight at 4°C, washed, and incubated with patient plasma for 2 hours at room temperature (28). Plates were washed, incubated with secondary anti-mouse IgG antibody (Abcam), and developed with alkaline phosphatase (Bio-Rad). Optical density was read at 405 nm (Molecular Devices). For determining anti-SARS-CoV-2 S1 protein IgA and IgG levels in patient plasma, we used ELISA kits according to the manufacturer’s instruction (Euroimmune).

### Statistics

GraphPad Prism 8 software was used for statistical analysis. Mouse experiment data were analyzed using Student *t*-test. Statistically significant comparisons were represented by asterisks in figures: *P < 0.05; **P < 0.005; ***P < 0.001. Actual P-values were reported in figure legends.

### Study Approval

Written informed consent was obtained from subjects, and the study protocol was approved by the Committee for the Protection of Human Subjects. This study was conducted in accordance with the guidelines in the Declaration of Helsinki. For mouse studies, all methods and protocols were approved by the Animal Care and Use Committee of the Johns Hopkins University.

## Results

### Case Presentation

**Figure 1:**
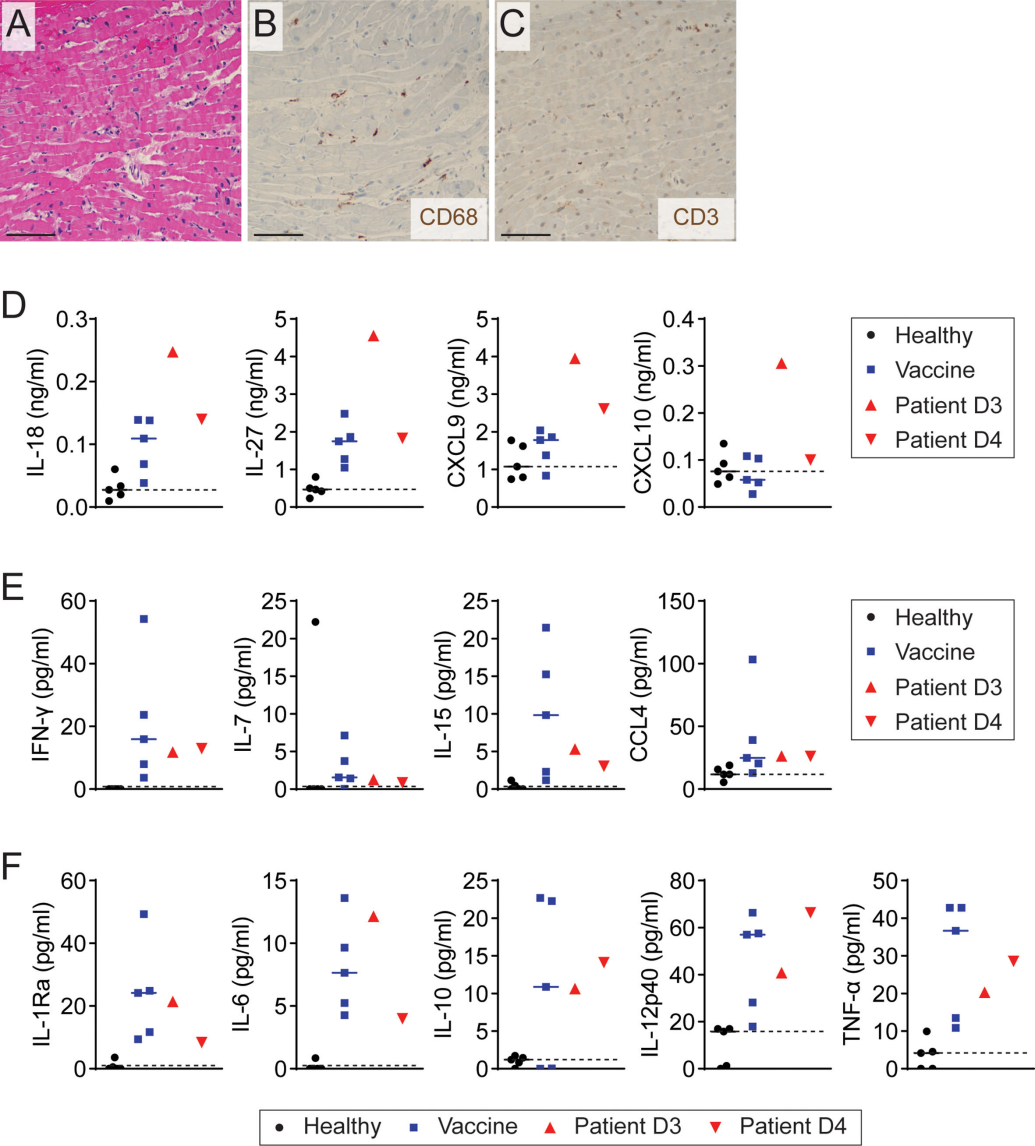
Elevation of Th1-type cytokines and chemokine in patient with myopericarditis after COVID-19 vaccination. **(A)** Representative image of H&E staining on endomyocardial biopsy from the patient with COVID-19 vaccine-related myopericarditis. Biopsy was collected on day 3 after COVID-19 mRNA vaccine. Scale bar: 12.5 µm. **(B, C)** Representative images of immunohistochemistry staining for CD68 **(B)** and CD3 **(C)** on endomyocardial biopsy of the patient. Scale bars: 12.5 µm. **(D–F)** Plasma cytokine and chemokine levels measured by multiplex cytokine assay in the patient with myopericarditis on days 3 (D3) and 4 (D4) after COVID-19 mRNA vaccination. As controls, five healthy controls (Healthy) and five individuals who had been vaccinated in the past 2 to 3 days (Vaccine) were tested.

A 24-year-old man with active tobacco use and asthma developed acute-onset substernal chest pain approximately 24 hours after receiving his first dose of the mRNA-1273 COVID-19 vaccine prompting presentation to the hospital (**Table 1**). Approximately three months before the vaccination, he had a mild infection with SARS-CoV-2 not requiring hospitalization. He had no history of drug abuse, inflammatory comorbidities, or recent vaccination. His ECG was notable for sinus rhythm with dynamic inferolateral ST elevations and anterior ST depressions. The initial serum cardiac troponin I level was 0.47 ng/ml (day 2 after vaccination), peaking at 46.0 ng/mL the day after admission (day 3 after vaccination). His nasopharyngeal SARS-CoV-2 NAT PCR was negative. He also tested negative for HIV and other respiratory viruses such as adenovirus, chlamydia pneumoniae, influenza A/B, metapneumovirus, mycoplasma pneumoniae, parainfluenzae 1-4, rhino/enterovirus, and RSV. He underwent emergent coronary angiography that showed no significant coronary artery disease or evidence of coronary vasospasm. Chest CT angiography was also negative for pulmonary embolism. Transthoracic echocardiogram showed mildly decreased left ventricular systolic function with ejection fraction 45-50%, mild to moderate mitral regurgitation, and moderate tricuspid regurgitation. Endomyocardial biopsy was performed on day 3 after the vaccination and revealed diffuse mild CD68^+^ cell infiltration with neither substantial inflammatory cell infiltration nor acute cardiomyocyte necrosis (**Figures 1A–C**). CMR showed mildly reduced left ventricular systolic function without late gadolinium enhancement. The nuclear antibody screen was negative. His clinical presentation was consistent with myopericarditis, and he was initiated on colchicine treatment with improvement and resolution of chest pain symptoms over the next two days. On discharge, he was prescribed metoprolol succinate and losartan and advised on exercise restrictions. Follow-up echocardiography at approximately three months demonstrated recovery of left ventricular systolic function (60-65%) and no other abnormalities (**Table 1**).

### Elevated Plasma IL-18 and Other Th1-Type Soluble Factors in Myopericarditis After COVID-19 Vaccine

To explore the involvement of immune system activation in myopericarditis after COVID-19 mRNA vaccination, we simultaneously measured 48 cytokines, chemokines, and growth factors in the patient plasma collected on days 3 and 4 after vaccination, depicted as D3 and D4 in the figures, using multiplex cytokine assay (Milliplex Map, EMD Millipore). As a control, we used two groups: five healthy males and five males who received the first dose of another mRNA vaccine, BNT162b2 (Pfizer-BioNTech), in the past 2 to 3 days, experiencing no cardiac complications or other side effects. In multiplex assay analysis, a biologically relevant change was considered if the patient sample showed an increase or decrease of more than two folds compared to a median value of either control group. We found that Th1-type cytokines IL-18 and IL-27 were increased in the patient with COVID-19 vaccine-related myopericarditis compared to both healthy and vaccine control groups (**Figure 1D** and **Supplementary Table 1**). Recently vaccinated controls also showed increased IL-18 and IL-27 levels compared with healthy controls, but they were lower than those of the patient sample collected on day 3 after the vaccination. Similarly, Th1-type chemokines, CXCL9 and CXCL10, were elevated in the patient with COVID-19 vaccine-related myopericarditis compared to healthy and vaccine controls (**Figure 1D** and **Supplementary Table 1**). The vaccine controls exhibited a comparable level of CXCL9 and CXCL10 to healthy controls. The patient with COVID-19 mRNA vaccine-associated myopericarditis presented increased levels of other Th1-type cytokines and chemokines (IFN-γ, IL-7, IL-15, and CCL4), pro-inflammatory cytokines (IL-6, IL-12p40, and TNF-α), and anti-inflammatory cytokines (IL-1Ra and IL-10) compared with healthy controls (**Figures 1E, F** and **Supplementary Table 1**). However, those cytokine levels in the patient were comparable to recently vaccinated controls. Thus, these data indicate that the patient with myopericarditis following COVID-19 vaccination had excessive Th1-type immune responses over vaccine-induced immune activation.

### No Contribution of Th2 and Th17 Immune Responses to Myopericarditis Following COVID-19 Vaccine

In the multiplex cytokine assays, plasma levels of Th2-related soluble factors such as IL-4, IL-5, IL-13, and CCL22 were comparable between the patient with COVID-19 mRNA vaccine-related myopericarditis and healthy controls (**Supplementary Table 1**). Unlike the patient, recently vaccinated controls showed an elevated level of Th2-type cytokines compared to the healthy control group (**Supplementary Table 1**). Eotaxin levels, a potent chemokine for eosinophil recruitment, were decreased in the patient with myopericarditis and vaccine controls compared with the healthy group (**Supplementary Table 1**). Meanwhile, the patient with myopericarditis after COVID-19 vaccination showed a slightly increased level of Th17-type cytokines including IL-17A, IL-17F, and IL-22 compared to healthy controls, but they were lower than those of recently vaccinated controls (**Supplementary Table 1**). These findings indicate that Th2- or Th17-dependent immune activation unlikely contributed to myopericarditis after COVID-19 vaccination.

### Activated Circulating NK Cells and T Cells in Myopericarditis After COVID-19 Vaccination

**Figure 2:**
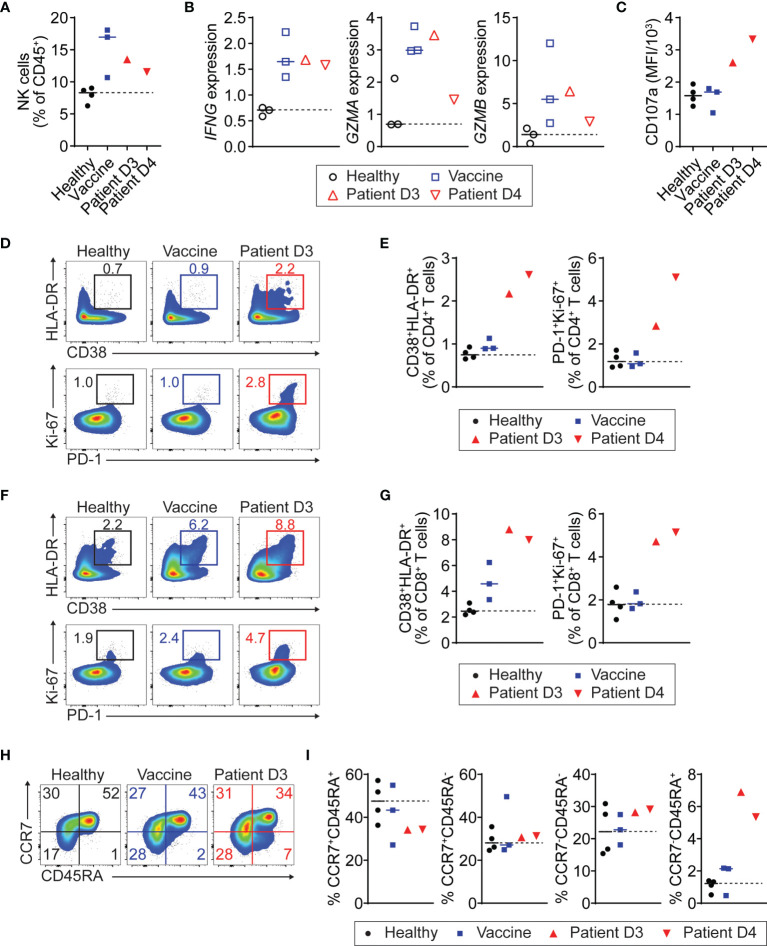
Activation of NK and T cells in patient with myopericarditis following COVID-19 vaccination. PMBC was isolated from the patient with myopericarditis on days 3 (D3) and 4 (D4) after COVID-19 mRNA vaccination. For flow cytometry analysis, four healthy controls (Healthy) and three individuals who had been vaccinated in the past 2 to 3 days (Vaccine) were used as control. For RT-PCR, three healthy and three vaccinated individuals were used as control. **(A)** NK cell frequency among PBMC CD45^+^ cells in the patient with COVID-19 vaccine-related myopericarditis. NK cells were gated on CD45^+^CD3^-^CD19^-^CD56^+^ (**Supplementary Figure 1**). **(B)** *IFNG*, *GZMA*, and *GZMB* gene expression in NK cells from the patient with COVID-19 vaccine-related myopericarditis and controls determined by RT-PCR. NK cells were enriched from the PBMC using magnetic beads. **(C)** Mean fluorescence intensity (MFI) of CD107a expression in PBMC NK cells of the patient with COVID-19 mRNA vaccine-related myopericarditis. **(D, E)** Flow cytometry plots **(D)** and frequencies **(E)** of CD38^+^HLA-DR^+^ and PD-1^+^Ki-67^+^ cells in PBMC CD4^+^ T cells. CD4^+^ T cells were gated on CD45^+^CD3^+^CD4^+^ (**Supplementary Figure 1**). **(F, G)** Flow cytometry plots **(F)** and frequencies **(G)** of CD38^+^HLA-DR^+^ and PD-1^+^Ki-67^+^ cells in PBMC CD8^+^ T cells. CD8^+^ T cells were gated on CD45^+^CD3^+^CD8^+^ (**Supplementary Figure 1**). **(H, I)** Flow cytometry plots **(H)** and frequencies **(I)** of CD4^+^ T cell subsets in the patient PBMC defined by CCR7^+^CD45RA^+^ (T_N_; naïve), CCR7^+^CD45RA^-^ (T_CM_; central memory), CCR7^-^CD45RA^-^ (T_EM_; effector memory), and CCR7^-^CD45RA^+^ (T_EMRA_; effector memory expressing CD45RA).

To further investigate changes in the immune profile of the patient with COVID-19 vaccine-related myopericarditis, we characterized his PBMC collected on days 3 and 4 after the vaccination using flow cytometry and RT-PCR (**Supplementary Figure 1**). We used PBMC from healthy or recently vaccinated individuals as controls. In flow cytometry and RT-PCR analysis, a biologically important change was considered if the value of the patient sample was higher or lower compared to all individuals of either control group. Regardless of myopericarditis development, the COVID-19 mRNA vaccine caused an increase of NK cell number and gene expression associated with effector function such as IFN-γ (*IFNG*), granzyme A (*GZMA*), and granzyme B (*GZMB*) compared with healthy controls (**Figures 2A, B**). Notably, NK cell expression of degranulation marker CD107a (LAMP-1; lysosomal-associated membrane protein 1) in the patient with myopericarditis after COVID-19 mRNA vaccine was higher than that of recently vaccinated controls (**Figure 2C** and **Supplementary Figure 2A**). In circulating T cells, both CD4^+^ and CD8^+^ T cells in the patient with myopericarditis after COVID-19 vaccine exhibited an activated phenotype defined by CD38^+^HLA-DR^+^ and PD-1^+^Ki-67^+^ compared to healthy and vaccine control groups (**Figures 2D–G**). The number of CD4^+^ and CD8^+^ T cells was comparable between the patient and control groups (**Supplementary Figure 2B**). However, the patient with myopericarditis after COVID-19 vaccine exhibited an increase of terminally differentiated effector memory CD4^+^ T cells expressing CD45RA (T_EMRA_; CCR7^-^CD45RA^+^) compared with both healthy and vaccinated controls (**Figures 2H, I**). The composition of other CD4^+^ T cell subsets such as naïve T cells (T_N_; CCR7^+^CD45RA^+^), central memory T cells (T_CM_; CCR7^+^CD45RA^-^), and effector memory T cells (T_EM_; CCR7^-^CD45RA^-^) was comparable between groups (**Figures 2H, I**). In CD8^+^ T cells, the patient with myopericarditis following COVID-19 vaccine showed a trend of increased T_EMRA_ subset and a decrease of T_CM_ and T_EM_ subsets compared to healthy and vaccinated controls (**Supplementary Figure 2C, D**). Thus, circulating NK cells and T cells might be activated directly by elevated IL-18 and IL-27 in the patient with COVID-19 vaccine-related myopericarditis.

### Monocyte NLRP3 Inflammasome Activation and IL-18 Production in COVID-19 Vaccine-Related Myopericarditis

**Figure 3:**
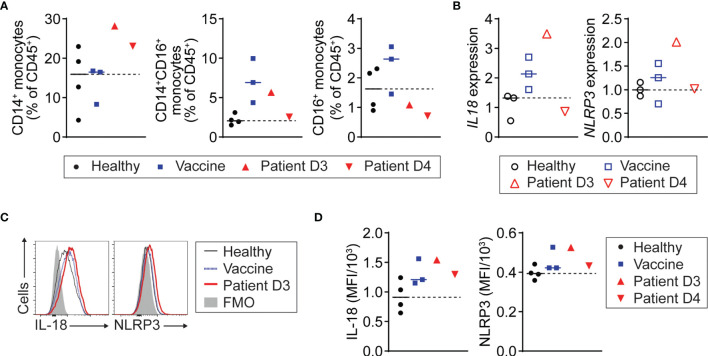
NLRP3 inflammasome activation and IL-18 production of monocytes in patient with COVID-19 vaccine-related myopericarditis. PMBC was isolated from the patient with myopericarditis on days 3 (D3) and 4 (D4) after COVID-19 mRNA vaccination. For flow cytometry analysis, four healthy controls (Healthy) and three individuals who had been vaccinated in the past 2 to 3 days (Vaccine) were used as control. For RT-PCR, three healthy and three vaccinated individuals were used as control. **(A)** CD14^+^, CD14^+^CD16^+^, and CD16^+^ monocyte frequency among PBMC CD45^+^ cells in the patient with myopericarditis following COVID-19 vaccine. Monocytes were gated on CD45^+^CD3^-^CD19^-^CD56^-^ (**Supplementary Figure 1**). **(B)** *IL18* and *NLRP3* gene expression in CD14^+^ monocytes from the patient with COVID-19 vaccine-related myopericarditis and controls determined by RT-PCR. CD14^+^ monocytes were enriched from the PBMC using magnetic beads. **(C, D)** Histograms **(C)** and mean fluorescence intensity (MFI) **(D)** of IL-18 and NLRP3 inflammasome expression in CD14^+^ monocytes of the patient with myopericarditis after COVID-19 mRNA vaccine.

Next, we tested whether monocytes in the patient with myopericarditis after COVID-19 vaccination are activated to produce IL-18. We found that the number of CD14^+^ classical monocytes was increased in the patient with myopericarditis after COVID-19 vaccination, while CD16^+^ non-classical monocytes were increased in the recently vaccinated controls (**Figure 3A**). CD14^+^ monocytes from the patient with myopericarditis on day 3 after the vaccination expressed a higher level of IL-18 (*IL18)* and NLRP3 inflammasome (*NLRP3*) genes than those of control groups (**Figure 3B**). Flow cytometry analysis confirmed increased intracellular IL-18 and NLRP3 inflammasome expression in CD14^+^ monocytes of the patient with COVID-19 vaccine-related myopericarditis (**Figures 3C, D**). Collectively, these findings suggest that blood monocytes in the patient with COVID-19 vaccine-related myopericarditis are activated to produce IL-18 priming NK cells and T cells.

### Mild Cardiac Dysfunction and NK Cell Activation in the Heart by IL-18 Treatment

**Figure 4:**
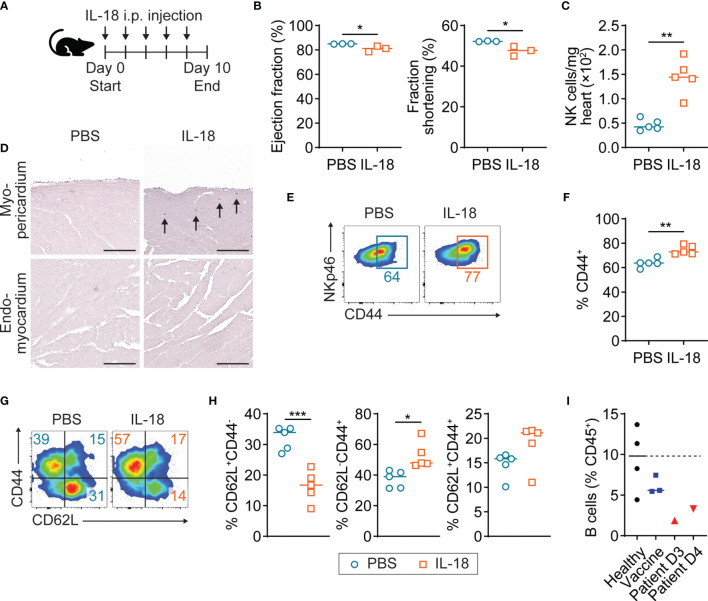
Mild cardiac dysfunction and NK cell activation in IL-18-treated mice. **(A)** Scheme of experimental timeline for IL-18 *in vivo* injection into A/J mice. Recombinant IL-18 was intraperitoneally injected into mice every other day for eight days. Mice underwent echocardiogram and then were sacrificed on day 10. **(B)** Ejection fraction and fraction shortening of IL-18-injected mice (n = 3) and controls (n = 3) monitored by M-mode echocardiogram. P-values: 0.0395 (left); 0.0255 (right). **(C)** Number of NK cells in hearts of IL-18-injected mice (n = 5) and controls (n=5) analyzed by flow cytometry. NK cells were gated on CD45^+^NKp46^+^ (**Supplementary Figure 3**). P-value: 0.0024. **(D)** Representative images of immunohistochemistry staining for NKp46 on the myocardium adjacent to the pericardium or endocardium from IL-18-injected mice. Arrows indicate NK cells. Scale bar: 25 µm. **(E, F)** Flow cytometry plots **(E)** and frequencies **(F)** of CD44 expression in cardiac NK cells of IL-18-injected mice. P-value: 0.0016. **(G, H)** Flow cytometry plots **(G)** and frequencies **(H)** of CD4^+^ T cell subsets defined by CD62L^+^CD44^-^ (naïve), CD62L^-^CD44^+^ (effector), and CD62L^+^CD44^+^ (memory) in IL-18-injected mouse hearts. CD4^+^ T cells were gated on CD45^+^NKp46^-^CD11b^-^CD3^+^CD4^+^ (**Supplementary Figure 3**). P-values: 0.0006 (left); 0.0104 (middle); 0.1089 (right). **(I)** Frequencies of B cells among PBMC CD45^+^ cells from the patient with COVID-19 vaccine-related myopericarditis. B cells were gated on CD45^+^CD19^+^ (**Supplementary Figure 1**). Student *t*-test was used for statistical analysis. *P < 0.05; **P < 0.005; ***P < 0.001.

In the endomyocardial biopsy of the patient with myopericarditis after COVID-19 vaccination, we observed diffuse mild infiltration of macrophages and monocytes, which can be a potent IL-18 producer in the heart (**Figure 1B**). Indeed, circulating monocytes in the patient with COVID-19 vaccine-related myopericarditis exhibited upregulated NLRP3 inflammasome expression and IL-18 production (**Figures 3B, D**). To investigate whether IL-18 can induce cardiac damage and inflammation, we injected recombinant IL-18 into mice and examined their cardiac function and immune profile (**Figure 4A**). In IL-18-injected mice, cardiac function monitored by ejection fraction and fraction shortening was slightly but significantly reduced compared to PBS-injected controls (**Figure 4B**). In cardiac infiltrating immune cell profiles, CD45^+^ cell number was increased in the IL-18-treated group compared with controls, although it was not statistically significant (**Supplementary Figures 3**, **4A**). No inflammatory cell foci were histologically observed in the myocardium or pericardium in IL-18-injected mice (data not shown). However, the number of cardiac NK cells was significantly increased in the IL-18-treated mice compared to controls (**Figure 4C**). Most NK cells in the IL-18-injected mouse hearts were located in the myocardium adjacent to the pericardium but not to the endocardium, suggesting a contribution of NK cells to myopericardial inflammation and damage (**Figure 4D**). Cardiac NK cells in the IL-18 treatment group exhibited increased CD44 expression, an activation marker, compared to controls (**Figures 4E, F**). The number of other immune cells in the heart such as T cells, B cells, macrophages, and monocytes was comparable between groups (**Supplementary Figure 4B**). Neutrophil counts were decreased in the hearts of IL-18-treated mice compared to controls (**Supplementary Figure 4B**). Despite no increase of cell numbers, cardiac CD4^+^ T cells in the IL-18-injected mice revealed an activated phenotype with significantly increased effector T cells (CD62L^-^CD44^+^) and decreased naïve T cells (CD62^+^CD44^-^) compared to controls (**Figures 4G, H**). Memory CD4^+^ T cells (CD62^+^CD44^+^) were also increased by IL-18 administration, although it was not significant (**Figures 4G, H**). CD8^+^ T cells in the hearts from IL-18-injected mice showed an activated phenotype as well as CD4^+^ T cells (**Supplementary Figure 4C, D**). Macrophages in the IL-18-treated mouse hearts were also activated, exhibiting increased MHCII expression (**Supplementary Figure 4E, F**). These data suggest that IL-18 can contribute to cardiac dysfunction directly or indirectly through the activation of NK cells and T cells, which were shown in the PBMC from the patient with myopericarditis after COVID-19 mRNA vaccine.

### No Involvement of Cardiac Autoantibodies or SARS-CoV-2 Infection in COVID-19 Vaccine-Associated Myopericarditis

To test if anti-cardiac autoantibodies are associated with myopericarditis following COVID-19 vaccine, we performed an indirect immunofluorescence assay using the patient plasma collected on days 3 and 4 after vaccination. We detected no significant antibody binding to the non-human primate heart tissue in the patient plasma, while positive control antibodies showed a strong binding (**Supplementary Figure 5A**). In addition, we tested if this patient had anti-cardiac myosin autoantibodies, which were present in patients with community-acquired myocarditis and inflammatory cardiomyopathy, by using ELISA (28). The patient had a negligible level of antibodies against cardiac myosin S2-16 and S2-28, comparable to normal healthy controls with no cardiac inflammation (**Supplementary Figure 5B**). In the PBMC of the patient with myopericarditis after vaccination, the number of B cells was reduced compared to healthy and vaccinated controls, while plasma cells were comparable between groups (**Figure 4I** and **Supplementary Figure 5C**). In the B cell subset analysis, naïve B cells (B_N_; IgD^+^CD27^-^), non-class-switched memory B cells (B_NSM_; IgD^+^CD27^+^), and class-switched memory B cells (B_SM_; IgD^-^CD27^+^) showed no proportional changes between the patient and control groups (**Supplementary Figure 5D**). The reduction of total B cell count in the patient with COVID-19 vaccine-related myocarditis could be temporarily caused by enhanced T cell immunity, not due to genetically defective humoral immunity. We found the presence of anti-SARS-CoV-2 spike (S) protein IgA and IgG in the patient plasma by ELISA (Euroimmun), while they were not detected in controls who received the vaccine at the same time point (**Supplementary Figure 5E**). In this patient, a history of COVID-19 three months before the vaccination likely induced anti-SARS-CoV-2 antibody production. Next, to investigate whether residual SARS-CoV-2 from the history of COVID-19 or cardiac S protein expression due to the mRNA vaccine is involved in myopericarditis in this patient, we tested the presence of SARS-CoV-2 S and nucleocapsid (N) genes in the endomyocardial biopsy by using RT-PCR. Neither viral gene was detected in the patient biopsy sample, suggesting no involvement of SARS-CoV-2 infection or cardiac S protein expression due to the vaccination in myopericarditis pathogenesis (data not shown).

## Discussion

We present a case of myopericarditis after the mRNA-1273 COVID-19 vaccine. Immune profiling of the patient plasma and PBMC revealed Th1-biased immune activation, especially highly elevated IL-18 levels and activated NK cells and T cells. In the endomyocardial biopsy, we found diffuse mild monocyte and macrophage infiltration. The patient presented with elevated cardiac troponin levels and mildly reduced cardiac function. In mice, IL-18 *in vivo* administration caused NK cell and T cell activation in the heart and mild cardiac dysfunction, replicating our findings in the patient with COVID-19 vaccine-related myopericarditis. Thus, a possible mechanistic explanation for myopericarditis after COVID-19 mRNA vaccine could be cardiac injury mediated by vaccination-triggered excessive IL-18 production and Th1 immunity activation as a consequence.

In the multiplex cytokine assay, the patient with myopericarditis after COVID-19 vaccination revealed a marked increase of four cytokines and chemokines, IL-18, IL-27, CXCL9, and CXCL10, over vaccinated controls who experienced no cardiac complications, suggesting that cardiac injury could be mediated by those soluble factors. This is supported by the finding of no significant inflammatory cell infiltration in the endomyocardial biopsy from the patient, where showed only diffuse macrophages and monocytes. In cardiovascular disease patients, high IL-18 levels were associated with a risk of heart damage and death, supported by animal studies (23–27). *In vitro* studies showed that IL-18 can directly induce cardiomyocyte hypertrophy and contractility reduction (27, 29). In addition, it was reported that NLRP3 inflammasome expression, an upstream signal for IL-18 activation, was upregulated in patients with myocarditis or pericarditis (30, 31). IL-27 is one of the cytokines elevated in patients with coronary artery disease, although it is unclear if IL-27 plays a pathogenic or protective role in the cardiovascular system (32–34). In the immune system, IL-27 plays a dual role by activating NK cells and Th1 cells and simultaneously promoting anti-inflammatory cytokine production in other types of T cells (35). CXCR3 ligands such as CXCL9 and CXCL10 are considered as biomarkers for heart failure and other cardiovascular diseases (36). CXCL9 treatment has disrupted electrophysiology in cardiomyocyte *in vitro* culture, while no studies have reported if CXCL10 can directly damage cardiomyocytes (37).

Th2-type cytokines and eotaxin play an essential role in eosinophilic myocarditis in humans and mice, and Th17-type immune responses are important for the pathogenesis of autoimmune myocarditis and its sequela dilated cardiomyopathy (38–43). In our study, we found that a level of Th2- or Th17-type cytokines in the patient with COVID-19 vaccine-related myopericarditis was comparable to healthy controls or lower than that of recently vaccinated controls. Thus, we concluded that Th2 cells, eosinophils, and Th17 cells are not involved in COVID-19 mRNA vaccine-related myopericarditis and that excessive Th1 immune activation might suppress Th2- and Th17-type immune responses in the patient with myopericarditis following the COVID-19 vaccine. In a case report by others, eosinophils within mixed inflammatory infiltration were observed in endomyocardial biopsy and autopsy specimens of patients with myocarditis after COVID-19 mRNA vaccination (12). However, those patients developed myocarditis at least 10 days after the vaccination, which is less typical than most cases reported and could suggest a different mechanism than the case in our report.

The patient with myopericarditis after COVID-19 mRNA vaccine revealed activated NK cells and T cells in the periphery, indicating a robust Th1-type immune response. Among elevated cytokines in the patients, IL-18 is a pro-inflammatory cytokine that can stimulate NK cells together with IL-12, and IL-27 is also known to augment NK cell effector function (44). In a similar way, IL-18 and IL-27 can activate T cells and differentiate them into Th1 cells (45). In the patient with myopericarditis following COVID-19 vaccination, we found an increase of monocyte number and IL-18 production. Monocytes are a primary source of IL-18 in the blood, constitutively expressing IL-18 mRNA and its membrane-bound form protein to release upon stimulation (46, 47). IL-27 can also be produced primarily by activated monocytes (48). In addition, we found diffuse myocardial CD68^+^ cell infiltration in the patient biopsy sample, suggesting an increased level of IL-18 produced by monocytes and macrophages in the heart with COVID-19 vaccine-related myopericarditis.

In our mouse experiment, IL-18 *in vivo* injection caused mild cardiac dysfunction. Previous clinical studies have suggested a detrimental role of IL-18 in various cardiovascular diseases (23–25). Supportively, other studies have shown that IL-18 can directly damage cardiomyocytes by inducing hypertrophy and reducing contractility (26, 27). Thus, elevated IL-18 production by circulating or heart-infiltrating monocytes and macrophages could directly cause cardiomyocyte injury in the patient with myopericarditis after COVID-19 vaccination, which was proven by high cardiac troponin levels and decreased left ventricular systolic function. Meanwhile, we found that IL-18 *in vivo* administration led to NK cell infiltration and activation in the myopericardium but not in the endomyocardium of mice, suggesting a contribution of NK cells to myopericardial inflammation. In myopericarditis after COVID-19 mRNA vaccine, a contribution of NK cells is unknown thus far. However, since NK cells are primarily and rapidly stimulated by IL-18, activated NK cells and their effector molecule production such as IFN-γ, perforin, granzymes, and FasL may contribute to myopericarditis onset and progress. A case report of myocarditis after the mRNA-1273 vaccine showed increased NK cell count in the patient blood as well as our finding (20). Due to well-known anti-viral function and Th1-type activity, NK cells have been considered to protect the heart from viral infection-induced myocarditis and eosinophil-associated autoimmune myocarditis in mice, but they are clinically different from COVID-19 vaccine-related myopericarditis (49, 50). Our mouse experiment showed that neutrophils were a minor population in the naïve heart, and their number was reduced after IL-18 treatment, suggesting no contribution of neutrophils to IL-18-driven cardiotoxicity. Supportively, in case reports by others, patients with myocarditis after COVID-19 vaccine presented no increase of blood neutrophil count (8).

A case study reported that the BNT162b2 mRNA vaccine caused cytokine release syndrome including IL-18 level elevation in a colorectal cancer patient (51). However, it is unclear which component of the mRNA vaccine is related to NLRP3 inflammasome-IL-18 pathway activation leading to cardiac damage. Since SARS-CoV-2-infected patients showed activated monocyte NLRP3 inflammasome pathway and increased plasma IL-18 levels, S protein expression due to mRNA vaccination could cause IL-18 production in monocytes (52, 53). Like the patient in our study, individuals who recovered from COVID-19 showed robust activation of CD8^+^ T cells after the first dose of mRNA vaccine than persons with no history of COVID-19 (54). Meanwhile, an immune enhancement by lipid nanoparticles has been studied in vaccine-injected mice (55). In addition, other researchers reported that the lipid nanoparticles could stimulate the NLRP3 inflammasome pathway through toll-like receptors, suggesting a possible monocyte activation and IL-18 production caused by the lipid nanoparticle vehicle of mRNA vaccine (56, 57).

Given the fact that 16- to 29-year-old males showed a higher incidence of COVID-19 vaccine-related myocarditis than other groups, we speculate that young men could have a stronger IL-18 production and Th1 immune response compared to females, although this requires further investigation (3). In clinical studies of community-acquired pericarditis and myocarditis cases, 66% to 84% of patients were males, and they were significantly younger than their female counterparts (58–61). Male patients with acute pericarditis experienced more myocardial involvement (myopericarditis) than females (58). Additionally, males presented more frequent ST elevation and higher troponin levels than females (58, 59). Similarly, in the mouse model for myocarditis induced by coxsackievirus B3 infection, male mice showed higher mortality and greater myocardial inflammation than females (62, 63). Cardiac IL-18 and IL-1β levels, the outcome of NLRP3 inflammasome activation, in male mice with myocarditis were significantly higher compared with females (63). A predominance of Th1-type immune response over Th2-type response was shown in the heart and periphery of male mice with myocarditis, suggesting a contribution of IL-18 and Th1 immune activation to sex-dependent cardiac inflammation (62–64).

Since COVID-19 mRNA vaccine-related myocarditis develops rapidly in 3 to 4 days after vaccination, innate immunity more likely contributes to the pathogenesis of vaccine-related myopericarditis than adaptive immunity (4). The pathogenic role of IL-1 family members such as IL-1α, IL-1β, and IL-33 in pericarditis has been studied clinically and preclinically. The therapeutic use of IL-1α and IL-1β inhibitors in patients with pericarditis has improved pain and inflammation in clinical trials (65). IL-1β treatment alone was able to cause cardiac dysfunction in mice, and IL-18 blockade by genetic mutation or inhibitor treatment prevented IL-1β-induced systolic dysfunction (66). This indicates that IL-18 is a potent mediator of cardiovascular diseases. In addition, we and others have shown that *in vivo* administration of IL-33 alone can induce acute pericarditis by activating only innate immunity in mice (67, 68). Thus, excessive activation of innate immune responses mediated by IL-18, an IL-1 family member, may contribute to myopericarditis development after COVID-19 mRNA vaccination. In IL-18-injected mice, we found NK cell infiltration in the myocardium near the pericardium, where inflammation or cardiomyocyte damage likely occurred in the patient with myopericarditis after COVID-19 mRNA vaccine. Future studies should explore whether IL-18 can damage the heart directly or indirectly through NK cell activation.

We found no autoantibodies against cardiac antigens associated with myocarditis in the patient with myopericarditis following COVID-19 vaccine. We also found neither SARS-CoV-2 S nor N gene expression in the endomyocardial biopsy of the patient. Molecular mimicry was also speculated, but we observed no significant T cell infiltration in the myocardium of the patient. The main limitation of this study is the small sample size and the higher age of the vaccinated control group.

## Data Availability Statement

The original contributions presented in the study are included in the article/**Supplementary Material**. Further inquiries can be directed to the corresponding author.

## Ethics Statement

The studies involving human participants were reviewed and approved by Committee for the Protection of Human Subjects at the Johns Hopkins University. The patients/participants provided their written informed consent to participate in this study. The animal study was reviewed and approved by Animal Care and Use Committee of the Johns Hopkins University.

## Author Contributions

TW, NG, and DC designed the study and wrote the manuscript. NG, JL, and AM collected biological and clinical data. CS was a cardiac pathologist in this case. TW, MW, DH, and MT performed experiments and analyzed data. All authors reviewed and approved the manuscript.

## Funding

This study was supported by the Matthew Vernon Poyner Memorial Foundation to DC and NG, the Bentivoglio Family Fund to NG, and the Post-Acute COVID-19 Syndrome Discovery Fund of the Johns Hopkins University to NG. NG is supported by the American Heart Association (AHA) 20SFRN35380046 and National Institutes of Health/National Heart, Lung, and Blood Institute (NIH/NHLBI) R01HL118183. DC is supported by NIH/NHLBI R01HL118183 and R01HL136586 and AHA COVID-19 Rapid Response Grant 814664, 20TPA35490421, and 19TPA34910007. TW is supported by the 2018 Rhett Lundy Memorial Research Fellowship from the Myocarditis Foundation. MW is funded by the NIH/National Institute of Arthritis and Musculoskeletal and Skin Diseases (NIAMS) F31AR077406. DH is supported by the NIH/NHLBI F31HL149328.

## Conflict of Interest

The authors declare that the research was conducted in the absence of any commercial or financial relationships that could be construed as a potential conflict of interest.

## Publisher’s Note

All claims expressed in this article are solely those of the authors and do not necessarily represent those of their affiliated organizations, or those of the publisher, the editors and the reviewers. Any product that may be evaluated in this article, or claim that may be made by its manufacturer, is not guaranteed or endorsed by the publisher.
