# Supplementary material for: Increased Interleukin 18-Dependent Immune Responses Are Associated With Myopericarditis After COVID-19 mRNA Vaccination
Source: Front Immunol. 2022 Feb 18;13:851620. doi: 10.3389/fimmu.2022.851620 (PMC8894592; doi:10.3389/fimmu.2022.851620)
Supplement: Supplementary file 1 [file DataSheet_1.pdf]

Supplementary Table 1. Multiplex cytokine analysis of the plasma from patient with myopericarditis after COVID-19 vaccine

Soluble factors (ng/ml)	Healthy controls (n=5, median)	Vaccine controls (n=5, median)	Patient day 3 (n=1)	Patient day 4 (n=1)
**Increased in patient compared to both healthy and vaccine controls**				
IL-18	27.32	109.18	247.54	139.98
IL-27	467.30	1747.30	4556.30	1826.40
CXCL9 (MIG)	1077.30	1780.28	3951.15	2608.25
CXCL10 (IP-10)	75.54	57.95	305.83	99.95
**Increased in patient compared to healthy controls at a same level as vaccine controls**				
IFN-γ	0.00	15.92	11.82	12.91
IL-1Ra	0.00	24.16	21.42	8.34
IL-6	0.00	7.65	12.15	3.99
IL-7	0.00	1.55	0.99	0.86
IL-10	1.21	10.88	10.64	14.10
IL-12p40	15.80	56.99	40.82	66.33
IL-15	0.00	9.84	5.31	3.03
TNF-α	4.16	36.64	20.31	28.53
CCL4 (MIP-1β)	11.68	24.93	26.42	26.19
**Increased in patient compared to healthy controls but lower than vaccine controls**				
IFN-α	0.00	43.30	2.70	0.00
IL-1α	0.00	197.11	26.46	17.89
IL-1β	0.00	8.26	0.19	0.00
IL-2	0.00	1.14	0.31	0.26
IL-8	1.27	23.45	2.09	3.12
IL-9	0.00	161.22	74.45	72.69
IL-12p70	0.00	4.82	0.49	0.61
IL-17A	0.00	2.93	0.42	0.39
IL-17E/IL-25	205.36	3759.12	437.43	171.12
IL-17F	0.00	1679.68	62.38	65.94
IL-22	0.00	230.45	28.36	25.52
TNF-β	0.00	17.82	0.91	0.91
CCL3 (MIP-1α)	0.00	85.27	16.01	21.37
CCL7 (MCP-3)	0.00	45.48	12.05	17.76
CXCL1 (GROα)	2.38	25.45	3.02	2.43
EGF	0.00	589.59	9.92	7.43
FGF-2	0.00	250.29	55.14	61.12
G-CSF	0.00	18.84	7.95	0.41
M-CSF	0.00	645.58	113.32	13.65
PDGF-AA	100.75	11535.95	440.48	118.87
PDGF-AB/BB	1821.33	86247.27	5663.03	2826.79
TGF-α	0.00	116.01	5.11	5.38
VEGF-A	0.00	75.60	6.23	3.48
sCD40L	0.00	545.34	121.03	63.51
**Decreased in patient compared to healthy controls but higher than vaccine controls**				
Eotaxin	343.23	58.13	154.25	150.70
**No difference between patient and healthy controls**				
IL-3	0.00	0.00	0.00	0.00
IL-4	0.00	1.44	0.00	0.00
IL-5	2.61	7.01	1.59	1.38
IL-13	0.00	14.52	0.00	0.00
CCL2 (MCP-1)	409.14	669.43	449.84	404.66
CCL5 (Rantes)	791.12	3777.68	1273.50	870.09
CCL22 (MDC)	717.81	1304.05	592.89	571.76
CX3CL1 (Fractalkine)	24.59	101.29	43.79	46.08
FLT-3L	20.75	131.10	30.86	31.42
GM-CSF	0.00	0.00	0.00	0.00

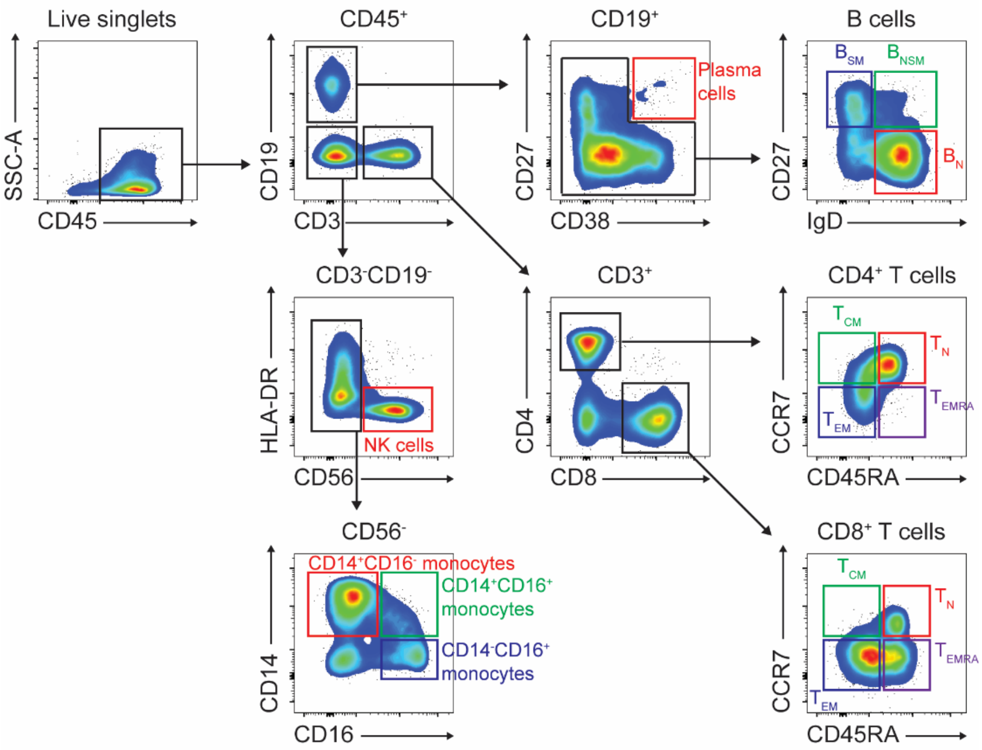

Supplementary Figure 1. Gating strategy for flow cytometry analysis of patient PBMC.

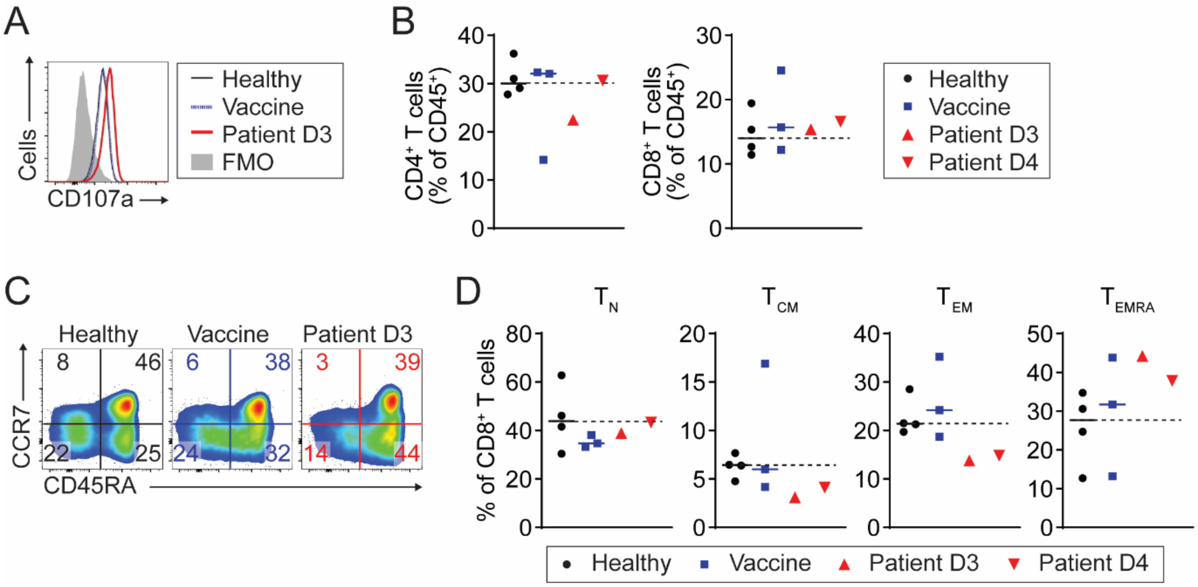

Supplementary Figure 2. Activated circulating T cells in patient with myopericarditis after COVID-19 mRNA vaccine. PMBC was isolated from the patient with myopericarditis on days 3 (D3) and 4 (D4) after COVID-19 mRNA vaccination. For flow cytometry analysis, four healthy controls (Healthy) and three individuals who had been vaccinated in the past 2 to 3 days (Vaccine) were used as control. For RT-PCR, three healthy and three vaccinated individuals were used as control. **(A)** Histograms of CD107a expression in PBMC NK cells of the patient with COVID-19 mRNA vaccine-related myopericarditis. **(B)** $CD4^+$ and $CD8^+$ T cell frequencies among PBMC $CD45^+$ cells in the patient with COVID-19 vaccine-related myopericarditis. $CD4^+$ T cells were gated on $CD45^+CD3^+CD4^+$, and $CD8^+$ T cells were gated on $CD45^+CD3^+CD8^+$ (Supplementary Figure 1). **(C,D)** Flow cytometry plots **(C)** and frequencies **(D)** of $CD8^+$ T cell subsets in the patient PBMC defined by $CCR7^+CD45RA^+$ (T_N; naïve), $CCR7^+CD45RA^-$ (T_{CM}; central memory), $CCR7^-CD45RA^-$ (T_{EM}; effector memory), and $CCR7^-CD45RA^+$ (T_{EMRA}; effector memory expressing CD45RA).

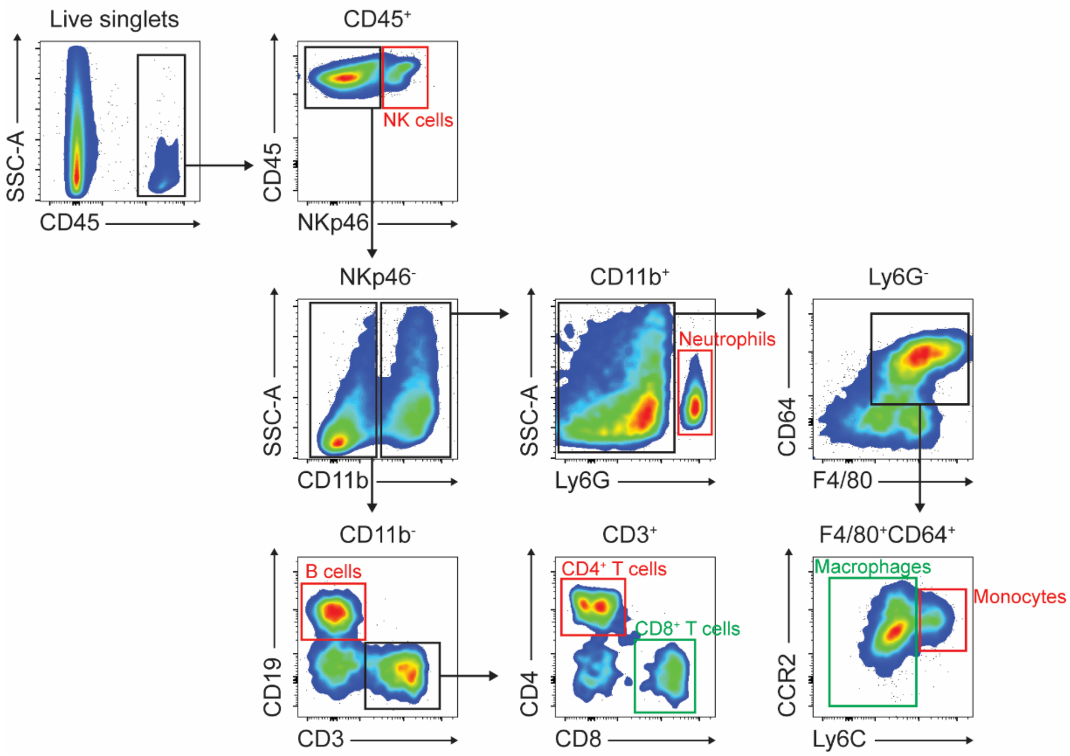

Supplementary Figure 3. Gating strategy for flow cytometry analysis of cardiac infiltrating immune cells from IL-18-injected mice.

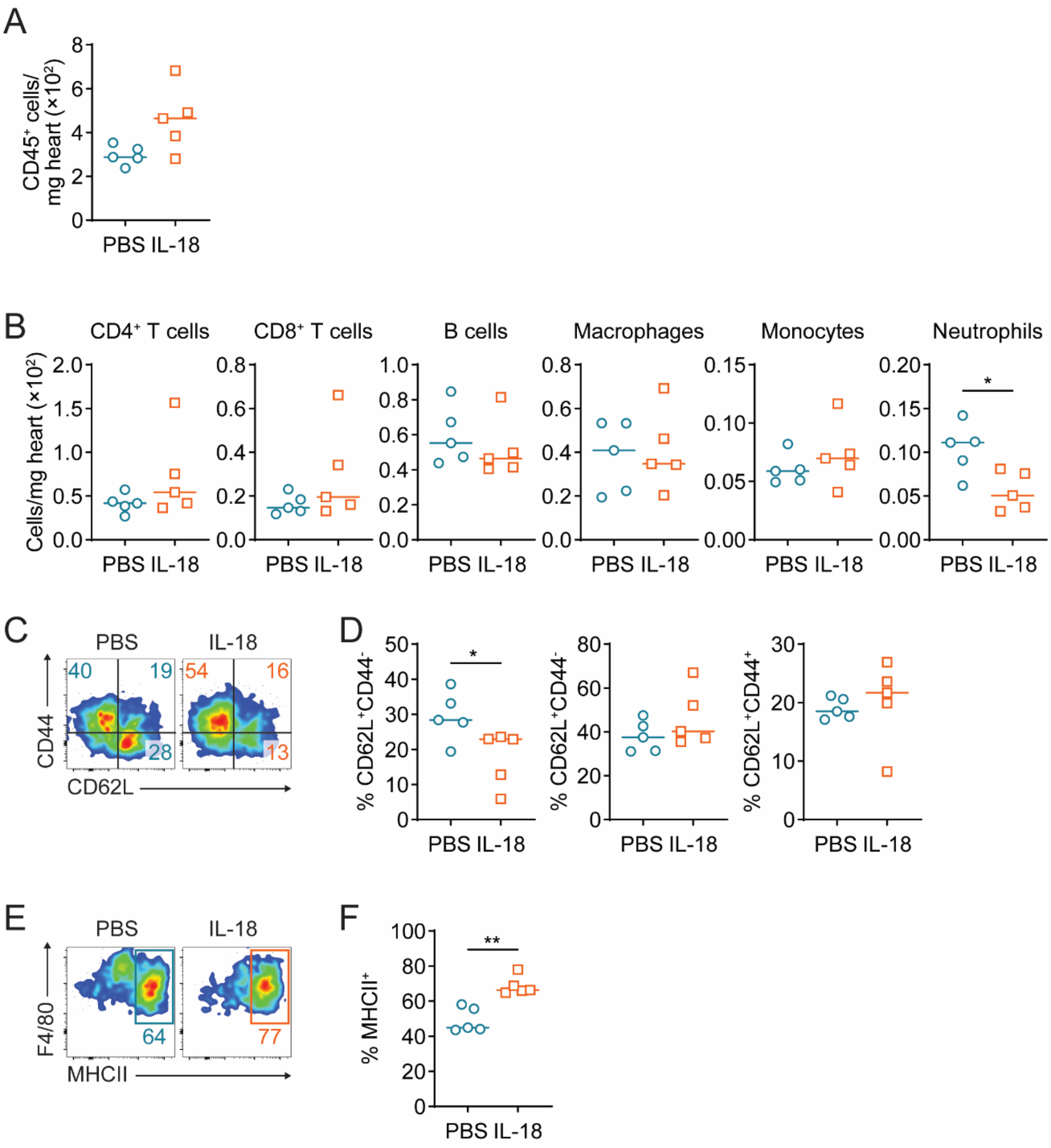

Supplementary Figure 4. Activated cardiac T cells and macrophages in IL-18-injected mice. Mice were intraperitoneally injected with recombinant IL-18 (n=5) or PBS (n=5) every other day for eight days. Mice were sacrificed on day 10. **(A)** Number of $CD45^+$ infiltrating cells in hearts of IL-18-injected analyzed by flow cytometry. P-value: 0.0687. **(B)** Number of $CD4^+$ T cells ($CD45^+NKp46^-CD11b^-CD3^+CD4^+$), $CD8^+$ T cells ($CD45^+NKp46^-CD11b^-CD3^+CD8^+$), B cells ($CD45^+NKp46^-CD11b^-CD19^+$), macrophages ($CD45^+NKp46^-CD11b^+Ly6G^-F4/80^+CD64^+Ly6C^-$), monocytes ($CD45^+NKp46^-CD11b^+Ly6G^-F4/80^+CD64^+Ly6C^+CCR2^+$), and neutrophils ($CD45^+NKp46^-CD11b^+Ly6G^+$) in hearts of IL-18-injected mice. P-value: 0.0196 (neutrophil). **(C,D)** Flow cytometry plots **(C)** and frequencies **(D)** of $CD8^+$ T cell subsets defined by $CD62L^+CD44^-$ (naïve), $CD62L^-CD44^+$ (effector), and $CD62L^+CD44^+$ (memory) in IL-18-injected mouse hearts. P-value: 0.0385 (left). **(E,F)** Flow cytometry plots **(E)** and frequencies **(F)**

of MHCII expression on macrophages in IL-18-injected mouse hearts. P-value: 0.0011. Student *t*-test was used for statistical analysis. *$P < 0.05$; **$P < 0.005$.

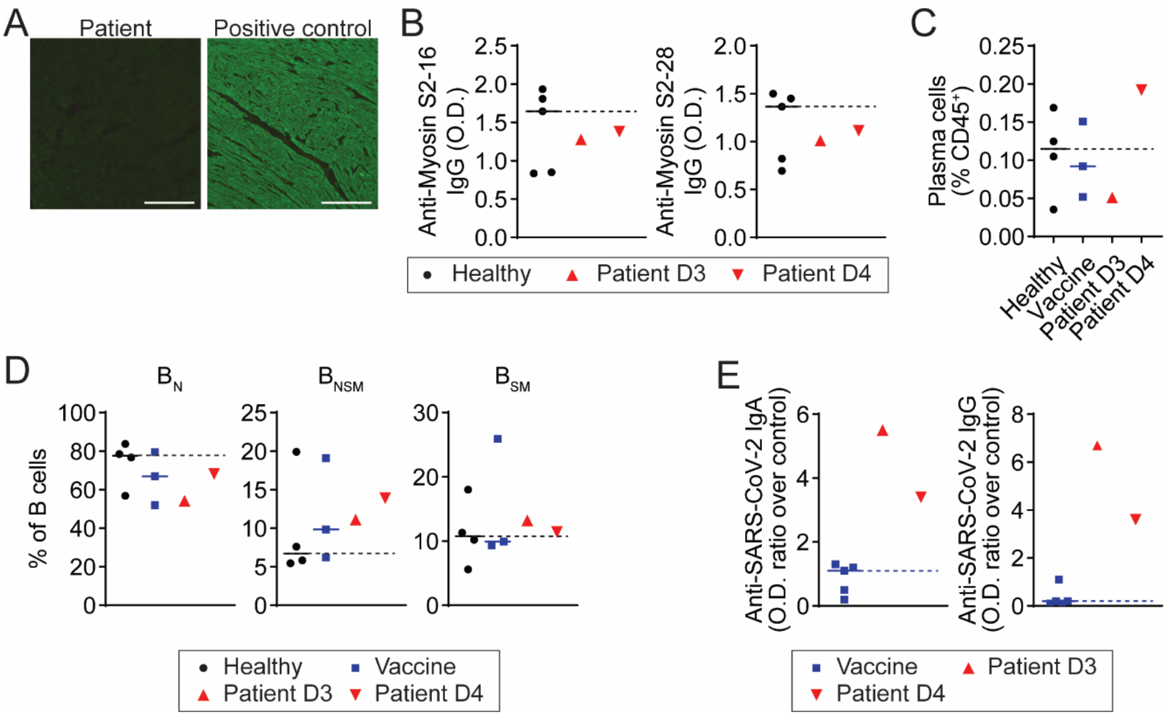

Supplementary Figure 5. No involvement of autoantibodies B cells in COVID-19 vaccine-related myopericarditis. **(A)** Representative image of indirect immunofluorescence assay for detecting anti-cardiac autoantibodies in the plasma of the patient with COVID-19 vaccine-related myopericarditis. The plasma was collected on day 3 after COVID-19 mRNA vaccine. Scale bars: 200 μm. **(B)** Plasma levels of antibodies against myosin peptide S2-16 or S2-28 measured by ELISA in the patient with myopericarditis on days 3 (D3) and 4 (D4) after COVID-19 vaccination. As controls, five healthy controls (Healthy) were tested. **(C)** Frequencies of plasma cells among PBMC $CD45^+$ cells from the patient with COVID-19 vaccine-related myopericarditis. Plasma cells were gated on $CD45^+CD19^+CD38^+CD27^+$ (Supplementary Figure 1). As controls, four healthy controls (Healthy) and three individuals who had been vaccinated in the past 2 to 3 days (Vaccine) were tested. **(D)** Frequencies of PBMC B cell subsets from the patient with COVID-19 vaccine-related myopericarditis defined by IgD^+CD27^- (B_N; naïve), IgD^+CD27^+ (B_{NSM}; non-class-switched memory), and IgD^-CD27^+ (B_{SM}; class-switched memory). B cells were gated on $CD45^+CD19^+$ (Supplementary Figure 1). **(E)** Plasma levels of IgA and IgG against SARS-CoV-2 spike protein measured by ELISA in the patient with myopericarditis on days 3 (D3) and 4 (D4) after COVID-19 vaccination. As controls, five controls who had been vaccinated in the past 2 to 3 days (Vaccine) were tested.
